# DCAN: Dynamic Channel Attention Network for Multi-Scale Distortion Correction

**DOI:** 10.3390/s25051482

**Published:** 2025-02-28

**Authors:** Jianhua Zhang, Saijie Peng, Jingjing Liu, Aiying Guo

**Affiliations:** Shanghai Key Laboratory of Chips and Systems for Intelligent Connected Vehicle, School of Microelectronics, Shanghai University, Shanghai 200444, China; jhzhang@shu.edu.cn (J.Z.); psj-yyds@shu.edu.cn (S.P.)

**Keywords:** distortion correction, dynamic channel attention network (DCAN), channel attention and fusion selective module (CAFSM), structural similarity loss (SSIM Loss)

## Abstract

Image distortion correction is a fundamental yet challenging task in image restoration, especially in scenarios with complex distortions and fine details. Existing methods often rely on fixed-scale feature extraction, which struggles to capture multi-scale distortions. This limitation results in difficulties in achieving a balance between global structural consistency and local detail preservation on distorted images with varying levels of complexity, resulting in suboptimal restoration quality for highly complex distortions. To address these challenges, this paper proposes a dynamic channel attention network (DCAN) for multi-scale distortion correction. Firstly, DCAN employs a multi-scale design and utilizes the optical flow network for distortion feature extraction, effectively balancing global structural consistency and local detail preservation under varying levels of distortion. Secondly, we present the channel attention and fusion selective module (CAFSM), which dynamically recalibrates feature importance across multi-scale distortions. By embedding CAFSM into the upsampling stage, the network enhances its ability to refine local features while preserving global structural integrity. Moreover, to further improve detail preservation and structural consistency, a comprehensive loss function is designed, incorporating structural similarity loss (SSIM Loss) to balance local and global optimization. Experimental results on the widely used Places2 dataset demonstrate that DCAN achieves state-of-the-art performance, with an average improvement of 1.55 dB in PSNR and 0.06 in SSIM compared with existing methods.

## 1. Introduction

Fisheye lenses are widely used in computer vision and digital image processing applications, including video surveillance systems [1,2], autonomous driving [3,4,5], VR devices [6,7], and robotics applications [8,9], due to their ability to capture a wider field of view compared with conventional cameras. However, fisheye lenses introduce significant challenges in the form of image distortion, particularly radial distortion. This distortion results in objects near the image center being overstretched, while the image periphery becomes overly compressed, leading to a loss of critical visual information. Consequently, fisheye images cannot be directly utilized for downstream tasks such as pose estimation [10,11,12], scene segmentation [13,14,15], and object recognition [16,17,18]. Therefore, correcting distortion in fisheye images is essential as a preprocessing step for geometric vision applications that rely on fisheye lenses.

Traditional vision calibration methods rely on calibration objects like checkerboards or geometric patterns, combined with multi-view images, to determine camera distortion parameters [19,20,21]. However, their dependence on such setups limits their practicality, particularly in real-time or dynamic environments [22,23]. To address these limitations, researchers have developed feature-based calibration methods that leverage scene geometry for automatic distortion correction. Thormahlen et al. [24] proposed a line-based calibration approach that eliminates the need for calibration objects or intrinsic parameters by removing outliers caused by true 3D curves, enhancing the robustness and accuracy of radial distortion estimation. However, this method is primarily suited for scenes with prominent linear features. Wang et al. [25] introduced a circularity-based correction method, which directly computes distortion parameters using the circular properties of distorted points, expanding the applicability to more diverse scenes while improving flexibility and robustness. Later, Bukhari et al. [26] developed a vertical-line-based method, integrating a division model and robust parameter estimation to achieve fast and precise radial distortion correction, particularly effective in structured environments. These approaches bypass the need for calibration objects by relying on inherent geometric features like lines and arcs, significantly improving their adaptability in dynamic or calibration-free scenarios. However, in natural scenes with sparse geometric features, detection errors may accumulate, potentially impacting the accuracy of distortion correction.

In recent years, deep learning has made significant progress in distortion correction, with convolutional neural networks (CNNs) emerging as the preferred approach due to their strong capability in local feature extraction. In 2017, Rong et al. [27] proposed a CNN-based radial distortion correction method that outperformed traditional baseline methods. The following year, Bogdan et al. [28] introduced a fully automated camera calibration method capable of regressing focal length and distortion parameters using only single images of regular scenes. Yin et al. [29] developed an end-to-end multi-context deep network that significantly improved fisheye distortion correction by integrating semantic-level and appearance-level feature learning. These parameter-based supervised learning methods directly map distorted parameters to their undistorted counterparts, demonstrating strong performance in controlled environments. However, they are highly sensitive to parameter estimation errors, where even minor inaccuracies accumulate across the image, leading to residual distortions. This sensitivity limits their robustness and precision, particularly in scenarios requiring high accuracy. To address these limitations, generative methods have emerged, leveraging deep neural networks to directly produce rectified images without explicit parameter estimation, exhibiting greater robustness and flexibility. For instance, Liao et al. [30] proposed a multi-level framework that decomposes the correction task into three stages: structural recovery, semantic embedding, and texture rendering, progressively generating the corrected image. Similarly, Yang et al. [31] introduced a dual diffusion architecture (DDA) that improves generalization by gradually introducing noise. Xu et al. [32] proposed a multi-scale progressive fusion strategy that demonstrated significant optimization potential in image registration tasks, inspiring its application in distortion correction to progressively capture global and local features, thereby improving correction accuracy and detail reconstruction. Compared with parameter regression methods, generative approaches exhibit stronger robustness and flexibility. However, they still face challenges in effectively incorporating selective attention during feature representation, which impacts global consistency and local detail preservation. Furthermore, many existing methods lack effective selective attention mechanisms [33], making it difficult to balance global consistency and local refinement, particularly in scenarios with complex distortions.

**Figure 1:**
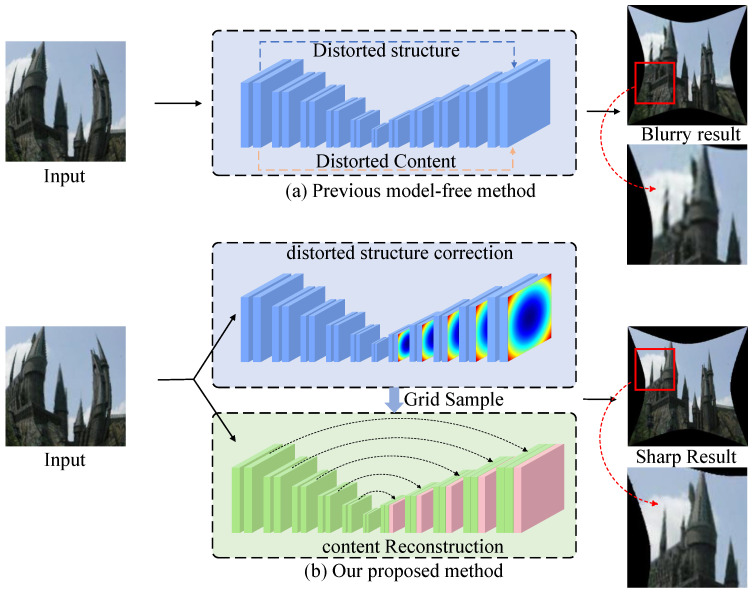
Comparison between previous single-scale correction methods and the proposed DCAN with multi-scale integration. (**a**) The previous single-scale-based method reconstructs distorted images directly from distorted features [34,35], resulting in blurry outputs and loss of structural details. (**b**) The proposed DCAN employs CAFSM to perform a multi-scale correction process, combining dynamic feature prioritization and selective fusion to refine both structure and content. By integrating predicted distortion features and correcting them through a cascade approach, DCAN achieves sharper results and better consistency in distortion correction.

To address the challenges of fisheye distortion correction, we propose the dynamic channel attention network (DCAN), which leverages a multi-scale framework to balance global structural consistency and local detail preservation. By integrating the channel attention and fusion selective module (CAFSM) into its architecture, DCAN dynamically prioritizes and refines features across multiple scales, enabling it to adapt to varying distortion levels effectively. Unlike traditional single-scale methods [34,35] that directly reconstruct images based on distorted features, often resulting in blurred and inconsistent outputs (Figure 1a), DCAN employs a multi-path progressive complementary mechanism to pre-correct distorted structures while refining content features. This cascaded correction approach (Figure 1b) ensures more accurate restoration and higher visual fidelity, even under complex distortion scenarios.

Here is a summary of our contributions:

- We propose the dynamic channel attention network (DCAN), which leverages a multi-path progressive complementary mechanism to effectively address the challenge of balancing global structural consistency and local detail preservation under varying distortion levels.
- We introduce the channel attention and fusion selective module (CAFSM), which dynamically prioritizes critical features and integrates multi-scale information, enhancing adaptability and feature representation of the network.
- We design a comprehensive loss function incorporating structural similarity loss (SSIM Loss) to ensure fine detail preservation and structural consistency, improving visual fidelity in the corrected images.

Section 2 reviews related work. Section 3 outlines the proposed DCAN architecture. Section 4 describes the experimental procedure and results. Finally, Section 5 concludes the paper and suggests future research directions.

## 2. Related Work

### 2.1. GAN-Based Distortion Correction Methods

In the field of distortion correction, researchers have begun to explore GAN-based frameworks to address challenges in rectifying distorted images. Liao et al. [35] further advanced this direction with DR-GAN, an end-to-end adversarial framework designed for radial distortion correction. DR-GAN bypasses the explicit parameter estimation process by directly learning the mapping between distorted and rectified images. This method significantly enhances flexibility and robustness in distortion correction tasks. However, like other GAN-based approaches, DR-GAN may face challenges in fine-grained detail reconstruction, particularly in scenarios involving severe or complex distortions. Chao et al. [36] proposed fisheye GAN (FE-GAN), which employs self-supervised learning and adversarial training to predict distortion flow and rectify fisheye distortions. By introducing geometric constraints such as cross-rotation consistency, FE-GAN effectively performs correction without requiring paired datasets or ground truth parameters. However, its reliance on pre-defined constraints limits its generalization to complex real-world distortions. To address these limitations, Yang et al. [37] introduced the progressively complementary network (PCN). By decomposing the correction process into multiple stages, PCN progressively refines distortion correction at different scales, improving both global consistency and local detail preservation.

### 2.2. Attention-Based Distortion Correction Methods

Attention-based methods leverage their ability to focus on significant features, enabling enhanced distortion correction by modeling global dependencies and capturing fine-grained details. For example, Guo et al. [38] proposed QueryCDR, which utilizes a query-based mechanism combined with attention modules to adaptively correct distortions at different levels. This approach improves flexibility and control by dynamically focusing on relevant features during correction. However, the added computational complexity of the query mechanism may limit its efficiency in real-time applications. Similarly, Feng et al. [39] introduced SimFIR, which integrates vision transformers (VIT) to capture multi-scale distortion features. By employing self-supervised learning, SimFIR enhances the representation of distortion patterns, contributing to improved downstream correction tasks. Despite its effectiveness in global feature modeling, the method lacks generative capabilities, limiting its ability to reconstruct fine-grained details in highly distorted images.

### 2.3. Integration of Channel Attention and Fusion Selective

Multi-scale feature integration plays a vital role in distortion correction tasks, as it balances global structural consistency with local detail refinement. Recent studies have explored feature integration techniques to enhance correction performance. Yang et al. [37] proposed the Progressively Complementary Network (PCN), which refines features at multiple stages, progressively improving the correction from coarse to fine. The network employs a recursive fusion mechanism, where each stage refines features by fusing information from previous stages. However, PCN lacks dynamic prioritization of key features, making it less adaptive in handling complex distortions with varying feature distributions. This limitation has also been addressed in earlier work on spatial feature selection [40].

In contrast, Hu et al. [41] proposed the squeeze-and-excitation (SE) module, which enhances feature representation by dynamically recalibrating channel weights. The module first reduces the global spatial information to channel descriptors and then generates adaptive weights for each channel. These recalibrated channel weights are applied to the feature map to refine the feature representation. Building on existing methods, we propose DCAN, which integrates the CAFSM into a multi-scale framework. This design addresses the limitations of previous approaches by dynamically prioritizing key features, combining progressive refinement with channel recalibration, and ensuring better adaptability to varying distortion levels.

## 3. Proposed Method

**Figure 2:**
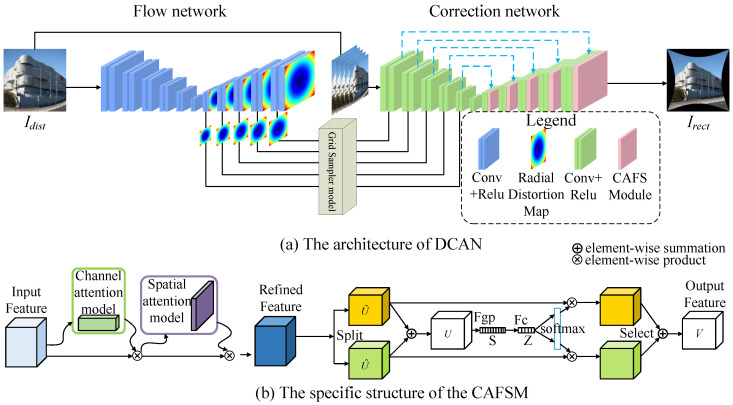
Overview network of the proposed DCAN method. (**a**) The architecture of DCAN. (**b**) The specific structure of the CAFSM.

In this section, we introduce the DCAN for multi-scale distortion correction. The overall architecture of the network is depicted in Figure 2, comprising three main components: an optical flow estimation module, a resampling module, and an aberration correction module. The implementation of DCAN is available at https://github.com/psj-yyds/fisheye_eye-correction.git, accessed on 6 January 2025. The optical flow estimation module is designed to extract and generate optical flow maps at multiple levels. By employing downsampling and upsampling operations, the module captures multi-scale features from the input fisheye image and produces optical flow maps that guide subsequent resampling and distortion correction. The resampling module adjusts pixel positions based on the generated optical flow maps, creating a pre-corrected image that reduces severe distortions. This intermediate output is then refined in the aberration correction module. A key innovation of the proposed method lies in the aberration correction module, where we introduce CAFSM. CAFSM enhances feature representation by dynamically prioritizing and integrating features across multiple scales. This integration effectively balances global structural consistency and local detail preservation, addressing the challenges posed by varying distortion complexities.

### 3.1. Flow Network

The optical flow estimation module utilizes an encoder–decoder framework, effectively extracting image features and producing a sequence of appearance flows, taking full advantage of this design. In the decoding phase, the module integrates a progressive complementarity mechanism, ensuring that the output from each decoder layer aids in generating the appearance flows. Notably, skip connections are established between encoder and decoder features, maintaining the precise spatial resolution. The encoder part consists of six hierarchical layers, each maintaining a 4 × 4 kernel size, with a stride of 2 (2×downsample), padding of 1, followed by a LeakyReLU activation function (α=0.2) and an instance normalization layer. The number of filters for each layer is as follows: 32, 64, 128, 256, 512, and 512.

The decoder comprises five hierarchical layers, each employing feature fusion, conventional convolutional processing, residual block enhancement, and transposed convolutional layers to progressively restore the spatial dimensions of the image and refine features. In detail, each layer begins by upsampling through a transposed convolutional layer, doubling the spatial dimensions while gradually reducing the number of channels to match the feature depth of the corresponding encoder layer. The first decoder layer halves the feature channels from 512 at the deepest layer to 256 and restores some spatial dimensions through a transposed convolutional layer. Subsequently, the output of each layer is fused with the output from the corresponding encoder layer, utilizing these cross-layer connections to enhance feature representation and supplement missing spatial information. The fused features are then further processed by conventional convolution, with residual blocks enhancing feature expression capability, followed by another transposed convolutional layer to continue enlarging spatial dimensions and reducing feature channels. This process is repeated across the decoder layers until the final layer reduces the channels to 16 while fully restoring the spatial dimensions to nearly match the input size. Before the final output, a positional layer combines the outputs of the decoder and the first encoder layer, generating the final two-channel flow map through convolution and residual blocks, accurately representing the movement direction of each pixel in the image. Ultimately, the module produces five appearance flows at different resolutions: 128, 64, 32, 16, and 8. The entire process can be represented as

(1) $${\left\{F_{i}\right\}}_{i=1}^{5}=F\left(I_{in}\right),$$

where $I_{in}$ stands for the input fisheye image, *F* refers to the optical flow estimation module, and $F_{i}$ represents the optical flow output from the *i*-th decoder layer.

### 3.2. Resample Network

The resampling module is a technique that adjusts the pixel positions of an image based on the optical flow field to generate a new image with specific transformation adjustments. This module generates a grid of coordinates for each pixel, adjusts these coordinates according to the optical flow field, and then resamples the original image using the modified coordinates to obtain the newly transformed image. The entire process can be represented as

(2) U={(i,j)∣i∈[0,W−1],j∈[0,H−1]},

where coordinate grid *U* is a matrix of W×H; each element *u* represents the pixel position (i,j) of the image. This grid undergoes a displacement adjustment by the optical flow field ΔU, which is generated hierarchically by the different layers of the optical flow estimation module.

This spatial transformation process can be expressed as: U′=U+ΔU. This equation can be expanded as

(3) $$U\prime =I_{i}\left(u+F_{i}\left(u\right),v+F_{i}\left(v\right)\right),$$

where $I_{i}$ represents the feature map of the *i*-th layer in the encoder–decoder module; u,v represent the horizontal and vertical coordinates in the coordinate grid; and $F_{i}\left(u\right),F_{i}\left(v\right)$ denote the displacement fields along the horizontal and vertical directions in the optical flow field, respectively. These displacement values are calculated based on the original pixel coordinates u,v and are used to compute the final displacement.

### 3.3. Correction Module

In the distortion correction module, the CAFSM operates on multi-scale feature maps ${\left\{F_{i}\right\}}_{i=1}^{5}$, extracted from the decoder layers. For each decoder layer, the input feature map $F_{\mathrm{input},i}$ is derived by applying a convolutional operation to the previous layer’s feature map, expressed as

(4) $$F_{\mathrm{input},i}=\mathrm{Conv}\left(F_{i-1}\right),$$

where $F_{i-1}$ is the feature map from the previous decoder layer. This hierarchical approach enables the module to leverage multi-scale features, progressively refining them at different levels for improved distortion correction.

To dynamically adjust feature importance, CAFSM employs a channel attention mechanism. For each feature channel, the global descriptor $z_{c,i}$ is computed as the spatial average of feature values across the height (*H*) and width (*W*):

(5) $$z_{c,i}=\frac{1}{H\times W}\sum_{u=1}^{H}\sum_{v=1}^{W}F_{c,i}\left(u,v\right),$$

followed by generating channel weights $s_{c,i}$ through two fully connected layers and activation functions

(6) $$s_{c,i}=\sigma \left(W_{2,i}\cdot \delta \left(W_{1,i}\cdot z_{c,i}\right)\right),$$

where σ(·) and δ(·) denote the Sigmoid and ReLU functions, respectively. The adjusted feature map ${\tilde{F}}_{c,i}$ is obtained by scaling the original map $F_{c,i}$ with $s_{c,i}$

(7) $${\tilde{F}}_{c,i}=s_{c,i}\cdot F_{c,i},$$

this mechanism enables the DCAN to focus on crucial channels while suppressing less relevant ones.

To enhance the integration of local and global features, CAFSM introduces a fusion selective mechanism. This mechanism adaptively combines refined local features ${\tilde{F}}_{i}$ and global features $F_{\mathrm{global},i}$ derived from all prior layers. The fused feature map $F_{\mathrm{fused},i}$ is calculated as

(8) $$F_{\mathrm{fused},i}=\alpha_{i}\cdot {\tilde{F}}_{i}+\beta_{i}\cdot F_{\mathrm{global},i},$$

where $\alpha_{i}$ and $\beta_{i}$ are learnable weights that balance the contributions of local and global information. The fused features are progressively integrated into the subsequent layers, with the updated input feature map at layer i+1 expressed as

(9) $$F_{\mathrm{input},i+1}=\mathrm{Fuse}\left(F_{\mathrm{fused},i},F_{\mathrm{fused},i-1}\right),$$

where Fuse(·) represents the fusion operation. Finally, the reconstructed feature map $\hat{F}$ is generated through an upsampling operation

(10) $$\hat{F}=\mathrm{Upsample}\left(F_{\mathrm{fused},L}\right),$$

where *L* denotes the last decoder layer. This progressive refinement effectively balances global consistency and local detail preservation, significantly improving the precision and robustness of distortion correction.

### 3.4. Training and Loss Function

To achieve superior performance in distortion correction, we optimize the DCAN with a comprehensive loss function designed to balance global structural consistency, local detail refinement, and multi-scale feature integration. This loss function combines three key components, each addressing a specific aspect of image correction. The overall loss function is formulated as

(11) $$L=\alpha_{S}L_{\mathrm{SSL}}+\beta_{E}L_{\mathrm{EHL}}+\gamma_{M}L_{\mathrm{MSL}},$$

where $L_{\mathrm{SSL}}$, $L_{\mathrm{EHL}}$, and $L_{\mathrm{MSL}}$ represent the SSIM Loss, Enhanced Loss, and Multi-Scale Loss, respectively. The hyperparameters $\alpha_{S},\beta_{E},\gamma_{M}$ are introduced to balance the contribution of each loss component during training. The rationale for incorporating these losses is detailed below.

SSIM Loss (SSL): To ensure the perceptual alignment of the output image $I_{\mathrm{out}}$ with the ground truth $I_{\mathrm{gt}}$, we adopt an enhanced SSIM Loss. This loss extends the traditional SSIM metric by incorporating dynamically computed channel weights $w_{c}$ and spatial feature intensities $f_{c}$, derived from the CAFSM. This design not only captures brightness, contrast, and structure but also dynamically prioritizes features based on their importance. The enhanced SSIM is expressed as

(12) $$SSIM_{\mathrm{enhanced}}\left(I_{\mathrm{out}},I_{\mathrm{gt}}\right)=\frac{\sum_{c=1}^{C}w_{c}\cdot f_{c}\cdot SSIM_{c}\left(I_{\mathrm{out}},I_{\mathrm{gt}}\right)}{\sum_{c=1}^{C}w_{c}\cdot f_{c}},$$

where $w_{c}$ represents channel-wise importance, and $f_{c}$ denotes spatial feature intensity. The SSIM Loss is computed as

(13) $$L_{\mathrm{SSL}}=1-SSIM_{\mathrm{enhanced}}\left(I_{\mathrm{out}},I_{\mathrm{gt}}\right),$$

by directly optimizing for structural similarity, this loss ensures that the generated images maintain both global structure and local detail fidelity.

Enhanced Loss (EHL): While the SSIM Loss captures perceptual quality, Enhanced Loss focuses on refining texture details and preserving structural accuracy. This loss combines content loss and style loss to balance pixel-level similarity and stylistic coherence

(14) $$L_{\mathrm{EHL}}=L_{\mathrm{Content}}+\lambda_{s}L_{\mathrm{Style}},$$

Content Loss: The Content Loss emphasizes feature-level alignment between the output and ground truth images. It leverages a pre-trained VGG network, which effectively captures high-level semantic and structural features beyond simple pixel-wise differences. Compared with traditional L1 or L2 loss, VGG-based content loss aligns better with human visual perception, ensuring that the corrected image preserves fine details and global consistency while reducing distortion. This is particularly beneficial for fisheye image rectification, where maintaining structural integrity is crucial. The loss is defined as

(15) $$L_{\mathrm{Content}}=\frac{1}{C_{j}H_{j}W_{j}}{∥\phi_{j}\left(I_{\mathrm{out}}\right)-\phi_{j}\left(I_{\mathrm{gt}}\right)∥}_{2}^{2},$$

where $\phi_{j}\left(x\right)$ represents the feature map from the *j*-th layer of the VGG network.

Style Loss: The Style Loss captures stylistic alignment by comparing the Gram matrices of the feature maps from the output and ground truth images

(16) $$L_{\mathrm{Style}}=\sum_{l}{∥G\left(\phi_{l}\left(I_{\mathrm{out}}\right)\right)-G\left(\phi_{l}\left(I_{\mathrm{gt}}\right)\right)∥}_{1},$$

here, G(·) denotes the Gram matrix of feature maps. By integrating both content loss and style loss, the enhanced loss ensures that the output images retain critical texture details and stylistic coherence.

Multi-Scale Loss (MSL): To leverage multi-scale information for comprehensive correction, we introduce the Multi-Scale Loss. This loss incorporates features at different resolutions, ensuring effective correction across both coarse and fine-grained scales. The formulation is

(17) $$L_{\mathrm{MSL}}=\sum_{i=1}^{L-1}{∥S\left(I_{\mathrm{gt}},i\right)-C\left(I_{c}^{i}⊕T_{d}^{i}\right)∥}_{1},$$

where S(x,i) resizes *x* to $1/2^{i}$ of its original size, and $I_{c}^{i}$, $T_{d}^{i}$ represent features from the decoder and correction layers, respectively. The operator ⊕ denotes feature concatenation.

Together, these three components form a robust framework for optimizing the network. The Structural Similarity Loss enhances perceptual quality, the Enhanced Loss refines structural and textural details, and the Multi-Scale Loss ensures that features from different resolutions contribute effectively to the final output. The hyperparameters $\alpha_{S},\beta_{E},\gamma_{M}$ are empirically tuned to maximize performance, demonstrating the efficacy of the proposed loss function in distortion correction tasks.

## 4. Experimental Procedure and Results

The evaluation of the proposed method is conducted using a synthetic fisheye dataset specifically designed to simulate diverse distortion scenarios. This dataset is generated based on the official Place2 dataset [42], which comprises over 10 million high-resolution images spanning more than 400 diverse scenes. To ensure sufficient variability for robust training and testing, one million images are randomly sampled for training, while 8000 images are reserved for testing. All images are resized to 256×256 pixels to match the input requirements of the model.

**Figure 3:**
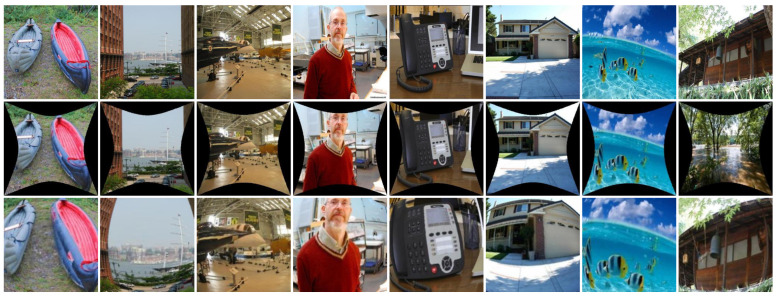
Synthetic fisheye dataset. (**Top**): Original images from Place2 dataset [42]. (**Middle**): Corrected images corresponding to the distorted samples. (**Bottom**): Generated fisheye images with different distortion levels.

The fisheye distortions are simulated using a polynomial distortion model parameterized by $Pd=[k_{1},k_{2},k_{3},k_{4}]$, defined as

(18) $$r_{d}=r_{u}\left(1+k_{1}r_{u}^{2}+k_{2}r_{u}^{4}+k_{3}r_{u}^{6}+k_{4}r_{u}^{8}\right),$$

where $r_{u}$ and $r_{d}$ represent the undistorted and distorted radii, respectively, relative to the image center. The distortion coefficients $k_{1}$, $k_{2}$, $k_{3}$, and $k_{4}$ control the degree of fisheye distortion, with higher-order terms capturing more complex distortion effects. In this study, the parameters are carefully selected within the ranges $k_{1}\in \left[10^{-4},10^{-6}\right]$, $k_{2}\in \left[10^{-9},10^{-11}\right]$, $k_{3}\in \left[10^{-14},10^{-16}\right]$, and $k_{4}\in \left[10^{-19},10^{-21}\right]$. As illustrated in Figure 3, this parameterized approach allows for the generation of a comprehensive dataset exhibiting a wide range of distortion intensities and patterns, supporting both model generalization and evaluation. By leveraging this dataset, the proposed method’s ability to handle varying levels of distortion is thoroughly assessed in both quantitative and qualitative experiments.

This parameterized approach allowed for the generation of a comprehensive dataset exhibiting a wide range of distortion intensities and patterns, supporting both model generalization and evaluation. By leveraging this dataset, the proposed method’s ability to handle varying levels of distortion is thoroughly assessed in both quantitative and qualitative experiments.

To provide a thorough validation of the proposed method, comparisons are conducted against a selection of state-of-the-art fisheye distortion correction approaches, including SimFIR [39], DeepCalib [28], DR-GAN [35], PCN [37], and QueryCDR [38]. These methods incorporate advanced neural network designs and attention mechanisms, representing a broad spectrum of strategies for addressing distortion correction. The evaluation protocol involved both quantitative metrics and qualitative visual assessments, ensuring a comprehensive analysis. Furthermore, to ensure consistency and fairness, all methods are trained and tested using the same dataset configuration.

The performance of DCAN is quantitatively evaluated using structural similarity (SSIM) [43], peak signal-to-noise ratio (PSNR) [44], and Fréchet Inception Distance (FID) [45]. These metrics are selected to comprehensively assess the structural consistency, pixel-level fidelity, and perceptual quality of the corrected images. Additionally, a complexity metric $C_{T}$ is introduced to classify the test dataset into different levels of difficulty, enabling a more detailed analysis of model robustness under varying scenarios. The mathematical definitions of the evaluation metrics are outlined below,

(19) $$\mathrm{SSIM}\left(x,y\right)=\frac{\left(2\mu_{x}\mu_{y}+C_{1}\right)\left(2\sigma_{xy}+C_{2}\right)}{\left(\mu_{x}^{2}+\mu_{y}^{2}+C_{1}\right)\left(\sigma_{x}^{2}+\sigma_{y}^{2}+C_{2}\right)},$$

where $\mu_{x},\mu_{y}$ are the mean intensities, $\sigma_{x},\sigma_{y}$ are variances, $\sigma_{xy}$ is the covariance, and $C_{1},C_{2}$ are stabilizing constants.

(20) $$\mathrm{PSNR}=10\cdot \log_{10}\left(\frac{{\mathrm{MAX}}^{2}}{\mathrm{MSE}}\right),$$

where MSE is the mean squared error and MAX is the maximum possible pixel value.

(21) $$\mathrm{FID}=∥\mu_{r}-\mu_{g}{∥}^{2}+\mathrm{Tr}\left(\Sigma_{r}+\Sigma_{g}-2{\left(\Sigma_{r}\Sigma_{g}\right)}^{1/2}\right),$$

where $\mu_{r},\mu_{g}$ and $\Sigma_{r},\Sigma_{g}$ are the means and covariances of the reference and generated images in the feature space.

Additionally, the complexity metric $C_{T}$ is defined as

(22) $$C_{T}=w_{1}\cdot \mathrm{KD}+w_{2}\cdot \mathrm{TV}+w_{3}\cdot \mathrm{ED},$$

where KD, TV, and ED represent keypoint density, texture variance, and edge density, respectively. Based on $C_{T}$, the test dataset is categorized into three complexity levels: low ($C_{T}<50$), medium ($50\leq C_{T}<100$), and high ($C_{T}\geq 100$).

The training process is carried out using the Adam optimizer, with $\beta_{1}=0.5$, $\beta_{2}=0.999$, and an initial learning rate of $10^{-4}$. To balance the contributions of different loss components in the overall objective function, the hyperparameters are empirically set to $\alpha_{S}=1.0$, $\beta_{E}=0.1$, and $\gamma_{M}=0.5$. These values ensure that the SSIM Loss ($L_{SSL}$), Enhanced Loss ($L_{EHL}$), and Multi-Scale Loss ($L_{MSL}$) collectively contribute to the optimization process while maintaining their respective impacts. Furthermore, learnable weights $\alpha_{i}$ and $\beta_{i}$ are introduced in the CAFSM to dynamically balance the integration of local and global features. Initially, these weights are set as 0.5 to ensure an equal contribution of local and global features at the start of training. These weights are updated dynamically through backpropagation and are constrained within the range [0,1], ensuring numerical stability and avoiding overfitting. This adaptive mechanism allows the model to prioritize features based on the characteristics of the input distortions, effectively improving the balance between global consistency and local detail preservation. By combining the carefully tuned hyperparameters and the dynamic adjustment mechanism of $\alpha_{i}$ and $\beta_{i}$, the proposed framework demonstrates superior adaptability and robustness across varying distortion levels.

The batch size is set to 32, and the model is trained for 180 epochs. The learning rate linearly decays from 0.2 to 0.05 over the training iterations. All experiments are conducted using the PyTorch framework (version 2.1.2) on an NVIDIA GeForce RTX 4090 GPU, ensuring efficient computation of the large-scale dataset.

### 4.1. Comparison Results

Quantitative Evaluation: The quantitative evaluation results, summarized in Table 1, present the PSNR, SSIM, and FID metrics across various complexity levels ($C_{T}<50$, $50\leq C_{T}<100$, $100\leq C_{T}<150$, $200\leq C_{T}<250$, $250\leq C_{T}<300$, and $C_{T}\geq 300$). These metrics comprehensively evaluate reconstruction accuracy, structural similarity, and perceptual quality, respectively. The dataset complexity is categorized into very low, low, medium, high, very high, and extreme levels based on keypoint density, texture variance, and edge density. In very low complexity scenarios ($C_{T}<50$), where distortions are minimal, the proposed method achieves a PSNR of **24.64** dB, exceeding the next-best performer, QueryCDR, by **1.32** dB. The SSIM of **0.9199** shows an improvement of **3.03%** compared with QueryCDR, while the FID of **27.7** indicates a perceptual quality enhancement over QueryCDR, which achieves a value of **28.3**. At low complexity levels ($50\leq C_{T}<100$), DCAN achieves a PSNR of **24.58** dB and an SSIM of **0.9159**, reflecting improvements of **1.62** dB and **5.71%** over QueryCDR and PCN, respectively. The FID of **27.8** further highlights the perceptual advantage, with QueryCDR recording a higher value of **28.9**. In medium complexity scenarios ($100\leq C_{T}<150$), DCAN achieves a PSNR of **24.43** dB, SSIM of **0.9191**, and FID of **28.1**. Compared with SimFIR, which records a PSNR of **21.84** dB and SSIM of **0.8411**, the proposed method provides improvements of **11.85%** and **9.27%**, respectively. The reduction in FID by **6.03%** compared with QueryCDR further demonstrates robustness in handling moderate distortions. For high complexity levels ($200\leq C_{T}<250$), the proposed method achieves a PSNR of **24.47** dB and an SSIM of **0.9195**, both outperforming QueryCDR, which achieves values of **22.79** dB and **0.7983**. The FID of **28.0**, which is lower than the value of **30.1** achieved by QueryCDR, highlights the ability of the proposed method to preserve perceptual fidelity in challenging conditions.At very high complexity levels ($250\leq C_{T}<300$), DCAN obtains a PSNR of **24.52** dB, SSIM of **0.9189**, and FID of **28.2**, maintaining a consistent advantage over competing approaches. When compared with SimFIR, the proposed method achieves an SSIM improvement of more than **10.37%**, demonstrating its effectiveness in preserving structural details even in scenarios with significant distortions. At the extreme complexity level ($C_{T}\geq 300$), the proposed method achieves a PSNR of **24.41** dB, SSIM of **0.9186**, and FID of **28.6**. These results exceed the corresponding values of QueryCDR, which records a PSNR of **22.86** dB, SSIM of **0.7895**, and FID of **30.9**, further validating the adaptability and robustness of the proposed method. Across all complexity levels, the proposed method attains an average PSNR of **24.51** dB, which is an improvement of **6.75%** over QueryCDR, with an average PSNR of **22.96** dB. Similarly, the average SSIM is **0.9187**, representing an enhancement of **6.98%** compared with PCN, which achieves an average SSIM of **0.8587**. The average FID of **28.1** is also superior to QueryCDR, which records a value of **29.9**, reflecting a perceptual improvement of **6.02%**. These results highlight the effectiveness of the advanced feature fusion mechanisms and structural preservation strategies employed by DCAN. Unlike fixed-pattern approaches such as QueryCDR, DCAN dynamically adjusts to varying levels of distortion, ensuring superior reconstruction accuracy and perceptual quality. This adaptability makes the proposed method well suited for real-world applications in diverse and challenging scenarios.

As shown in Table 2, DCAN achieves an effective balance between computational complexity and inference speed through multi-scale feature extraction and progressive refinement. While it has slightly higher FLOPs and parameters than lightweight architectures such as SimFIR and DeepCalib, it maintains competitive runtime efficiency, demonstrating its capability to process high-resolution distorted images effectively. This trade-off highlights the architectural optimization that ensures both computational efficiency and high-fidelity correction, making DCAN well suited for demanding distortion correction tasks.

**Figure 4:**
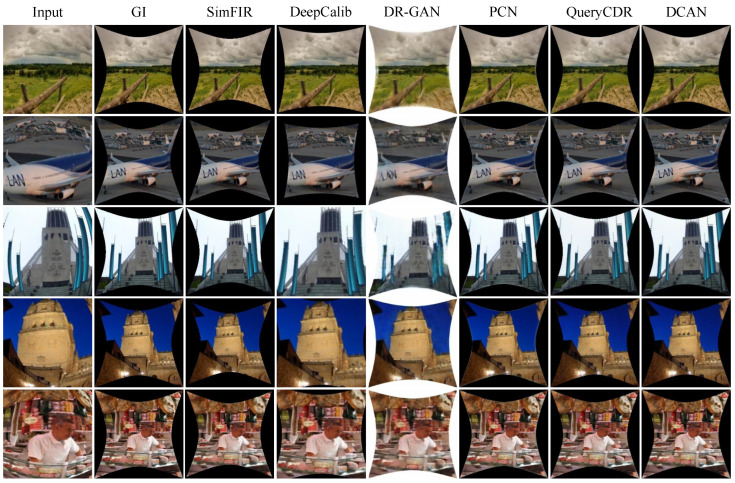
Comparison of synthetic images. Visual results comparison in different scenarios. The state-of-the-art methods include the regression-based method (DeepCalib) and four generation-based methods (SimFIR, DR-GAN, PCN, and QueryCDR).

**Figure 5:**
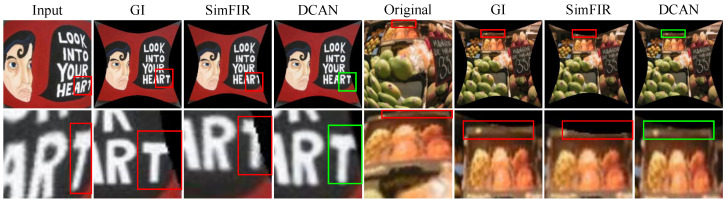
Additional comparison between SimFIR and DCAN. DCAN results provide additional information.The red borders (regions with distortion or unclear details) and the green borders (regions where DCAN improves clarity and preserves details) are used to highlight the improvements.

**Figure 6:**
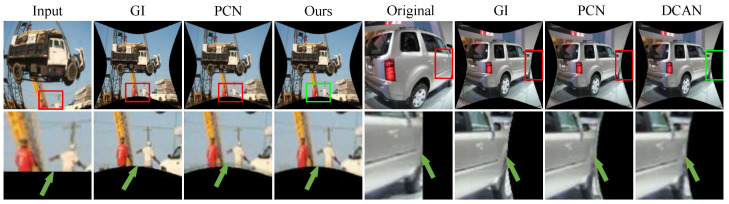
Additional comparison between PCN and DCAN. DCAN results provide additional details.The red borders (regions with distortion or unclear details) and the green borders (regions where DCAN improves clarity and preserves details) are used to highlight the improvements.

**Figure 7:**
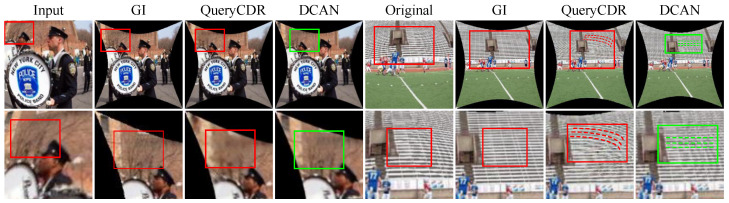
Additional comparison between QueryCDR and DCAN. DCAN results demonstrate notable performance in both detail correction and image clarity to a significant extent.The red borders (regions with distortion or unclear details) and the green borders (regions where DCAN improves clarity and preserves details) are used to highlight the improvements.

Qualitative Evaluation: In this section, we use our synthetic dataset to visualize the corrected photos of the various algorithms in order to provide a visual comparison. The learning methods SimFIR, DeepCalib, DR-GAN, PCN, and QueryCDR achieve improved correction performance in terms of visual appearance thanks to the benefits of the global semantic features supplied by neural networks, as Figure 4 illustrates. However, these approaches have difficulty recovering appropriate distributions from substantially distorted situations because of the simplicity and inadequacy of the learning methodology. The distorted parts of the image are corrected by SimFIR, although the ground truth picture of physical objects is often smaller and loses some edge information as a result. DeepCalib [28] corrects the inner areas of the picture well while degrading in the boundary regions. The network’s inherent properties impose limitations on DR-GAN and the images it generates show blurring. PCN frequently yields overcorrected results, which makes it difficult to flexibly adjust to varying degrees of distortion. Additionally, Figure 5, Figure 6 and Figure 7 provide a comprehensive comparison with SimFIR, PCN, and QueryCDR. The comparative results with SimFIR and PCN can be visually observed and clearly demonstrate the advantages of DCAN. To further highlight the differences between DCAN and QueryCDR, we introduce bounding boxes in the corrected images. Specifically, green boxes mark areas with high-quality correction results, while red boxes indicate regions where correction performance is less satisfactory. These visual comparisons effectively illustrate the strengths of DCAN in recovering fine details and achieving better correction consistency across the entire image. The results demonstrate that DCAN achieves significantly better correction quality compared with the other methods, highlighting its superior ability to retain fine details and avoid excessive smoothing. This indicates that DCAN not only effectively prevents information loss and blurring but also ensures a high level of detail recovery in the corrected images. Moreover, in qualitative evaluations, DCAN outperforms most of the compared approaches, achieving the best corrective performance and closely approximating the ground truth.

**Figure 8:**
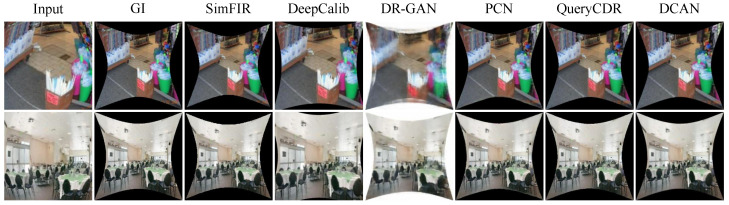
Qualitative comparison of real-world fisheye images. From left to right, we show the input distorted images, the results of five different methods (SimFIR, DeepCalib, DR-GAN, and PCN) and QueryCDR, and the results of our proposed method. Our method achieves the best overall visual quality among all compared methods.

Comparison of Real-world Images: To confirm the feasibility of the approach, we used artificial data to train the network. Subsequently, we test the generalization performance of DCAN using real-world fisheye photos, as Figure 8 illustrates. The figure shows that, despite the inevitable disparities between artificial and natural fisheye photos, the correction outcomes of DCAN are comparatively superior to previous approaches in terms of both global scene distribution and information retention. The outcomes demonstrate how effectively DCAN generalizes to fisheye photos found in the real world.

**Figure 9:**
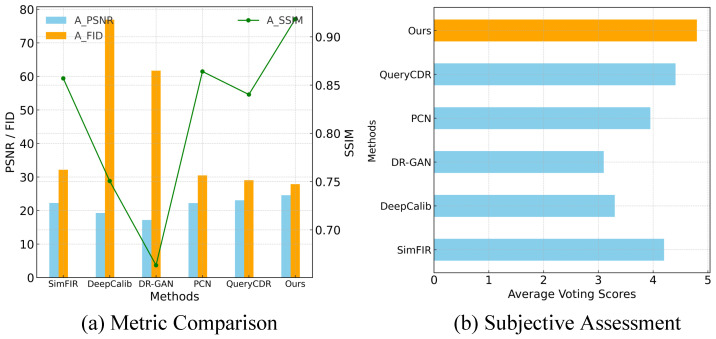
Metric comparison and subjective evaluation. (**a**) A comparison of the average PSNR, SSIM, and FID across different fisheye correction methods. (**b**) Subjective assessment based on voting scores for correction results.

To comprehensively evaluate the performance of different fisheye correction methods, both objective metrics and subjective assessments are utilized. Figure 9a presents the average performance of PSNR, SSIM, and FID across various methods, where higher PSNR and SSIM values indicate better image fidelity and lower FID scores reflect more realistic and visually consistent results. For subjective evaluation, a voting experiment is conducted involving ten volunteers with experience in image processing. A total of 200 fisheye images are randomly selected from diverse scenes, including campus, streets, and indoor environments, and are rectified using different methods. To ensure fairness, the images are divided into ten groups, each containing 20 images, with their order randomized to avoid bias. Each group is evaluated by a single volunteer, who rated the rectified images on a scale of 0 (worst) to 5 (best). All participants have a strong background in image processing and are familiar with the image content, ensuring reliable feedback. As shown in Figure 9b, DCAN achieved the highest subjective ratings among all approaches.

### 4.2. Ablation Study

Comprehensive ablation experiments are performed to assess the contributions of individual modules and loss functions to the overall performance of DCAN. This section specifically examines the influence of the CAFSM on the quality of the generated images. By systematically removing these components, we aim to elucidate their roles in the image restoration process.

Experimental analysis of structure and loss function ablation: Ablation studies are conducted on both the network structure and loss functions of DCAN to evaluate their respective contributions to the overall performance, as shown in Table 3. The removal of the flow estimation module (w/o FNM) results in substantial performance degradation, with PSNR decreasing to 15.88 dB, SSIM to 0.4713, and FID increasing to 198.7, highlighting its critical role in effective distortion correction. Similarly, the absence of the distortion correction module (w/o DCM) leads to improved results compared with w/o FNM, with PSNR and SSIM reaching 17.96 dB and 0.6434, respectively, though still significantly lower than the complete model. Furthermore, excluding the channel attention and selective fusion module (w/o CAFSM) reduces PSNR to 22.36 dB and SSIM to 0.8779, emphasizing its importance in enhancing correction capabilities. Regarding loss functions, the removal of all losses (w/o EHL and MSL and SSL) causes noticeable performance degradation, with PSNR, SSIM, and FID recorded at 22.38, 0.8688, and 31.9, respectively. The exclusion of structural similarity loss (w/o SSL) further reduces SSIM to 0.8772, underscoring its significance in preserving structural details. Similarly, removing enhancement and multi-scale losses (w/o EHL and MSL) results in PSNR and SSIM values of 23.87 dB and 0.8801, respectively. The complete model of DCAN, integrating all modules and loss functions, achieves the best performance, with a PSNR of 24.59 dB, an SSIM of 0.9110, and an FID of 28.6, validating the soundness of the proposed design and the complementary contributions of each component.

**Figure 10:**
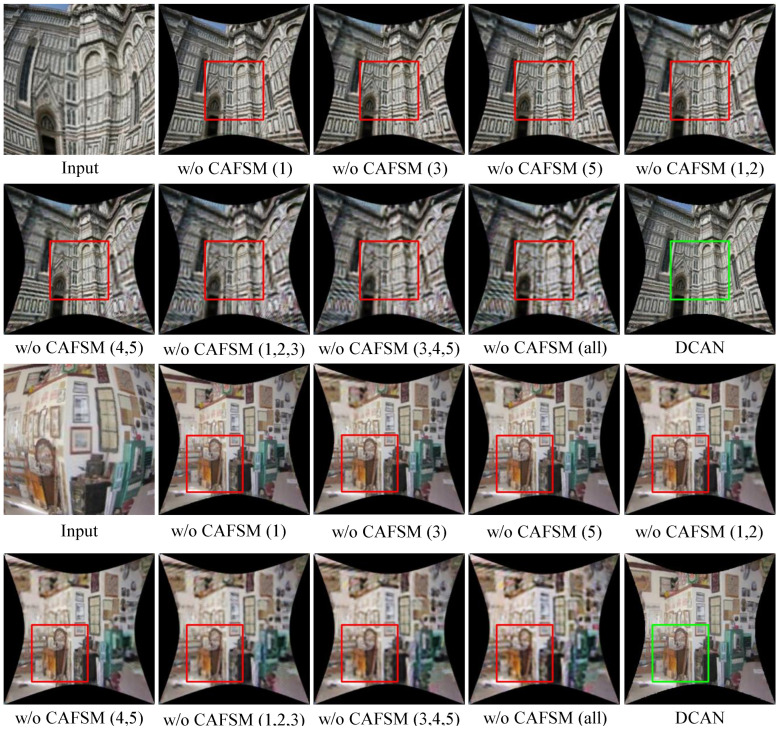
A comparison of results without CAFSM on different layers.

Experimental analysis of CAFSM ablation: To further explore the role of CAFSM in different convolutional layers, layer-by-layer ablation experiments are designed, and the results are shown in Table 4 and Figure 10. Firstly, removing the CAFSM in layer 1 (w/o CAFSM (1)) decreases the PSNR to 23.12 dB and the SSIM to 0.8600, indicating that the CAFSM in layer 1 is essential in correcting the external features. The performance decreases slightly when the CAFSM of layers 3 and 5 (w/o CAFSM (3) and CAFSM (5)) are removed, indicating that these layers also contribute to detail correction. Removing multiple CAFSMs (w/o CAFSM (1,2) and CAFSM (4,5)) results in a further decrease in PSNR and SSIM; in particular, the removal of all CAFSMs (w/o CAFSM (all)) results in a PSNR of only 22.36 dB and an SSIM of 0.8779. To further visualize the results of these ablation experiments, the corrected regions in the test images are highlighted using bounding boxes, where green boxes denote areas with high-quality correction, and red boxes indicate regions with noticeable degradation or suboptimal correction. This visualization method intuitively demonstrates the contribution of the CAFSM at different layers to the correction of specific image regions. Comparing the full model results shows that the CAFSM contributes positively to the final correction effect at every layer, especially at the middle and deep layers of the convolutional network. Thus, it is shown that the CAFSM plays an indispensable role in improving image correction accuracy and enhancing image detail processing capability. Through these ablation experiments, we further demonstrate the core value of the CAFSM in DCAN, which is crucial for the overall image correction effect and provides targeted optimization at different feature levels.

## 5. Conclusions

In conclusion, DCAN integrates a dedicated streaming network and a correction network augmented with the CAFSM, achieving improvements in addressing complex fisheye distortions. By combining channel and spatial attention mechanisms with a selective kernel fusion strategy, CAFSM enhances feature representation during up-sampling, enabling precise and visually superior corrections. The network effectively preserves essential details and ensures structural consistency across different image regions, demonstrating robustness and adaptability. Comprehensive experiments validate the proposed DCAN. The results demonstrate consistent outperformance of existing approaches across evaluation metrics, including an improvement of +1.5 dB in PSNR, +0.10 in SSIM, and −1.8 in FID when compared with the latest method, QueryCDR. These improvements highlight the dual-path architecture and the effectiveness of CAFSM in addressing challenging distortion scenarios while preserving fine details and structural integrity. Additionally, the modular design of the network provides flexibility for future enhancements and potential applications in broader image-processing tasks.

### Future Works

Future work will enhance the model’s robustness for extreme distortion and complex content while exploring domain adaptation. We will also optimize it for real-time applications like video correction and AR, ensuring stable performance on hardware accelerators. These efforts aim to expand DCAN’s use in autonomous driving and media.

## Figures and Tables

**Table 1:** Comparison across different complexity levels ($C_{T1}:C_{T}<50$, $C_{T2}:50\leq C_{T}<100$, $C_{T3}:100\leq C_{T}<150$, $C_{T4}:200\leq C_{T}<250$, $C_{T5}:250\leq C_{T}<300$, $C_{T6}:C_{T}\geq 300$) (bold indicates the best value).

**Methods**	**PSNR ↑**						
	$C_{T1}$	$C_{T2}$	$C_{T3}$	$C_{T4}$	$C_{T5}$	$C_{T6}$	**Avg**
SimFIR [39]	22.58	22.32	21.84	21.75	21.71	21.68	21.98
DeepCalib [28]	19.73	18.68	19.33	19.81	19.76	19.72	19.51
DR-GAN [35]	17.41	17.12	16.96	16.77	16.90	16.86	17.00
PCN [37]	22.48	22.32	21.88	21.59	21.73	21.71	21.95
QueryCDR [38]	23.32	22.96	22.87	22.79	22.95	22.86	22.96
DCAN	**24.64**	**24.58**	**24.43**	**24.47**	**24.52**	**24.41**	**24.51**
**Methods**	**SSIM**↑						
	$C_{T1}$	$C_{T2}$	$C_{T3}$	$C_{T4}$	$C_{T5}$	$C_{T6}$	**Avg**
SimFIR [39]	0.8735	0.8562	0.8411	0.8331	0.8325	0.8319	0.8447
DeepCalib [28]	0.8102	0.7285	0.7132	0.7565	0.7553	0.7541	0.7530
DR-GAN [35]	0.6996	0.6514	0.6389	0.6278	0.6331	0.6322	0.6472
PCN [37]	0.8837	0.8588	0.8496	0.8511	0.8552	0.8538	0.8587
QueryCDR [38]	0.8896	0.8312	0.7997	0.7983	0.7881	0.7895	0.8161
DCAN	**0.9199**	**0.9159**	**0.9191**	**0.9195**	**0.9189**	**0.9186**	**0.9187**
**Methods**	**FID**↓						
	$C_{T1}$	$C_{T2}$	$C_{T3}$	$C_{T4}$	$C_{T5}$	$C_{T6}$	**Avg**
SimFIR [39]	30.6	32.8	33.1	33.5	33.7	33.8	32.9
DeepCalib [28]	71.7	78.9	80.1	80.4	80.6	80.9	78.8
DR-GAN [35]	60.1	62.4	62.6	63.1	62.9	63.2	62.4
PCN [37]	29.4	30.8	31.2	31.3	31.1	30.9	30.8
QueryCDR [38]	28.3	28.9	29.9	30.1	31.2	30.9	29.9
DCAN	**27.7**	**27.8**	**28.1**	**28.0**	**28.2**	**28.6**	**28.1**

**Table 2:** Computational complexity and inference time comparison (bold indicates the best value, underline indicates the second-best value).

Methods	SimFIR [39]	DeepCalib [28]	DR-GAN [35]	PCN [37]	QueryCDR [38]	DCAN
Flops (G)	**2.98**	8.33	12.86	12.31	12.35	12.34
Parameters (M)	**11.64**	22.06	54.43	35.63	43.24	36.75
Time (s)	0.58	**0.43**	0.70	0.55	0.53	0.49

**Table 3:** Performance comparison of different structures and loss functions (bold indicates the best value).

	Methods	PSNR ↑	SSIM ↑	FID ↓
Network Module	w/o FNM	15.88	0.4713	198.7
	w/o DCM	17.96	0.6434	163.5
	w/o CAFSM	22.36	0.8779	32.7
Loss Function	w/o EHL and MSL and SSL	22.38	0.8688	31.9
	w/o SSL	23.11	0.8772	31.5
	w/o EHL and MSL	23.87	0.8801	29.1
DCAN	all	**24.59**	**0.9110**	**28.6**

**Table 4:** Performance of using channel attention and selective fusion modules on different convolutional layer outputs (bold indicates the best value).

Methods	PSNR ↑	SSIM ↑	FID ↓
w/o CAFSM (1)	23.12	0.8600	30.6
w/o CAFSM (3)	23.76	0.8713	30.4
w/o CAFSM (5)	23.95	0.8971	29.9
w/o CAFSM (1,2)	22.98	0.8598	31.2
w/o CAFSM (4,5)	23.78	0.8801	30.0
w/o CAFSM (1,2,3)	22.41	0.8581	31.5
w/o CAFSM (3,4,5)	23.85	0.8771	29.1
w/o CAFSM (all)	22.36	0.8779	32.7
DCAN	**24.59**	**0.9110**	**28.6**

## Data Availability

All datasets used are available online with open access.
